# The Hyper-IgE Syndromes: Lessons in Nature, From Bench to Bedside

**DOI:** 10.1097/WOX.0b013e31825a73b2

**Published:** 2012-07-15

**Authors:** Efren L Rael, Robert T Marshall, Jonathan J McClain

**Affiliations:** 1 Section of Allergy, Asthma and Immunology, Penn State, Milton S. Hershey Medical Center MCH0401, 500 University Drive, Hershey, PA 17033-2360; 2 Penn State, Milton S. Hershey Medical Center, School of Medicine, Hershey, PA

**Keywords:** STAT3, DOCK8, TYK2, T_H_17, Job syndrome, Hyper-IgE syndrome, immunodeficiency

## Abstract

Hyper-IgE syndrome is a primary immunodeficiency marked by abnormalities in the coordination of cell-cell signaling with the potential to affect T_H_17 cell, B cell, and neutrophil responses. Clinical manifestations include recurrent skin and lung infections, serum IgE elevation, connective tissue repair and development alterations, and the propensity for vascular abnormalities and tumor development. Signal transducer and activator of transcription 3 (STAT3) signaling, dedicator of cytokinesis 8 (DOCK8) signaling, and tyrosine kinase 2 (TYK2) signaling alterations have been implicated in 3 forms of hyper-IgE syndrome.

## Signal Transducer and Activator of Transcription 3 Signaling

The *STAT3 *gene, located on chromosome 17q21, encodes a transcription factor that dimerizes with itself, signal transducer and activator of transcription 1 (STAT1), or STAT4 after cognate receptor activation and translocates to the nucleus to affect transcription in response to multiple cytokines [1]. The interleukin (IL)-6 family members (IL-6, IL-1, IL-31, LIF, CNTF, CLC/CLF, NP, CT1, and OSM), IL-10 family members (IL-10, IL-19, IL-20, IL-22, IL-24, and IL-26), IL-12 family members (IL-23 and IL-27), IL-21, granulocyte colonystimulating factor, and leptin signal through STAT3 [2,3]. Furthermore, STAT3 has been implicated in G protein-coupled receptor signaling and in cellular homeostatic control mechanisms involving mitochondrial regulation of reactive oxygen species (ROS) generation [4,5]. As a result, *STAT3 *mutation has the potential for wide ranging effects on the immune system and metabolically active cells, including cancer cells.

**Figure 1:**
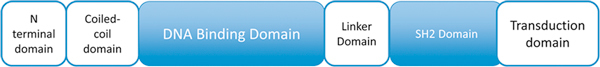
**Organization of the STAT3 protein**.

*STAT3 *mutations have been identified in the coiled-coil domain (important for interactions with other proteins), the DNA-binding domains (important for transcriptional regulation), the Src homology 2 (SH2) domains (important in Stat protein dimerization and receptor contacts), and the gene splice sites/trans activation sites (associated with protein interactions important in gene transcription)[6,7] (Figure 1). Dominant negative mutations in this gene lead to the autosomal dominant form of hyper-IgE syndrome (HIES) (AD-HIES) and a marked reduction in T_H_17 cells [7].

AD-HIES in vitro studies reveal variably impaired neutrophil responses marked by diminished neutrophil chemoattractant receptors FPR, CXCR1, and CXCR2 [8]. FPR is a high-affinity receptor for bacterial N-formyl peptides important for neutrophil chemotaxis and microbicidal activity [8]. CXCR1 and CXCR2 receptors are responsive to ELR+ CXC chemokine subfamily members, including CXCL1, 6, 7, and 8 [9-22]. In addition to neutrophil trafficking response alterations, signaling to neutrophils is impaired. Minegishi et al[23] demonstrated an inability of T cells to induce skin keratinocyte and bronchial epithelial cell recruitment of neutrophils with anti-staphylococcal molecules, such as CXCL8. Furthermore, a reduction in T_H_17 cells can skew adaptive, innate immune system communication responses to *Candida*. T_H_17 cytokines, IL-17 and IL-22, regulate antimicrobial peptides, such as histatins and β-defensin-2 [24]. Deficits of these antimicrobial peptides may help to explain why saliva from subjects with AD-HIES often has reduced candicidal activity than controls [24]. Additional innate immune response deficits in AD-HIES include altered toll-like receptor (TLR2) responses in the absence of T_H_17 cell versus TLR2 signaling in the presence of T_H_17 cell responses [23].

T_H_17 cell reduction associated with STAT3 deficiency might be a generalizable phenomenon specific to T-cell differentiation [25]. Siegel et al[25] demonstrated that AD-HIES subjects had decreased central memory CD4^+ ^and CD8^+ ^T cells and increased naive T cells, consistent with a proliferation and differentiation defect from naive precursors. These findings may corroborate with a propensity to develop varicella zoster virus reactivation in AD-HIES [25].

B-cell dysregulation associated with IgE elevation in HIES is incompletely understood. IL-21 signals through STAT1, STAT3, and STAT5. Studies with naive IL-21 receptor knockout mice demonstrate increased IgE levels but low IgG1 levels [26]. Human in vitro B-cell studies reveal alternative IL-21, context dependent, IgE regulation with low IgE levels in the context of IL-21 incubation with PHA and IL-4, but increased IgE in the context of IL-21 incubation with anti-CD40 and IL-4, suggesting that T-cell-B-cell pathway miscommunication may play a role in IgE elevation [27]. In addition to IgE alterations in AD-HIES, peripheral blood B lymphocytes are skewed toward transitional and naive cells with marked reduction in memory B cells independent of the STAT3 mutation location [28].

The percentage of peripheral blood memory B cells is frequently reduced in AD-HIES [29]. Furthermore, AD-HIES subjects have defects in peripheral blood memory B-cell subsets, characterized by multiparameter flow cytometry as CD38^low^CD24^hi ^[30]. Further characterization of cells from this AD-HIES cohort, in comparison with controls, demonstrated decreased numbers of all B-cell subsets, both switched and unswitched with the greatest decrease in the IgA+ switched and IgM+ unswitched memory B-cell populations [30]. Plasma analysis revealed that STAT3-HIES patients had a statistically significant increase in B-cell-activating factor of the TNF family (BAFF), a B-cell survival factor, expression versus controls, which correlated with a decrease in BAFF receptor expression [30]. Furthermore, a positive correlation between National Institutes of Health (NIH) HIES scores and plasma BAFF scores was identified [30]. Most subjects in this cohort also had poor T-cell dependent antibody responses to bacteriophage phiX174 primary and secondary immunizations [30]. The authors comment that these findings reflect both altered B cell-intrinsic and B cell-extrinsic STAT3 signaling defects [30].

Tissue remodeling proteins are altered in AD-HIES [31]. Matrix metalloproteinases (MMP)-8 (important for contribution to acute lung inflammation and tissue remodeling after acute lung injury) andMMP-9 (important for vascular smooth muscle cell migration, macrophage recruitment and elastin degradation) are elevated 3-fold during periods free from infection in AD-HIES versus controls [31]. Furthermore, MMP-3 (important for cardiovascular matrix remodeling) was lower in AD-HIES subjects versus control subjects [31].

In a mouse model, Stat3 inactivation in osteocytes and osteoblasts results in bone mechanical alterations, including decreased bone strength, mass, and load response generation, as well as impairment in ROS regulation [32].

These findings give further validation to previous studies suggesting that STAT3 plays a role in regulating cellular homeostasis through mitochondrial oxidative stress and ROS responses [4,33].

## Dedicator of Cytokinesis 8 Signaling

**Figure 2:**
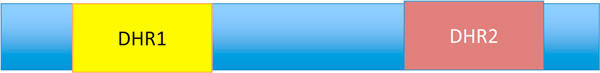
**Organization of the DOCK8 protein**.

*DOCK8 *is located on chromosome 9p24 [34]. Loss of dedicator of cytokinesis 8 (DOCK8) protein function leads to the most common form of autosomal recessive HIES (AR-HIES). DOCK8 is in the family of guanine exchange factors that can activate Rho GTPases, which include ras-related C3 botulinum toxin substrate 1 (RAC1) and cell division control protein 42 homolog (CDC42) [35]. Both RAC1 and CDC42 mediate important effects on actin cytoskeletal rearrangements, whereby CDC42 plays a role in effects at the leading edge of cell remodeling resulting in filopodial protrusions, whereas RAC1 affects lamellipodial protrusions [36]. Rho GTPase family members can affect STAT3 phosphorylation, directly or indirectly (however, these interactions in DOCK8-deficient patients have not been reported to the authors' knowledge) [37-39]. *Dock8 *deletions have been identified in the Dock homology region 1 (DHR1) domain [important for binding phosphatidylinositol (3,4,5)-triphosphate (PIP3) membrane-rich regions, which assist in perimembrane positional organization] and in the DHR2 domain (important for binding to Rho family GTPases)[40,41] (Figure 2). Hence, DOCK8, through interactions with Rho GTPases, may play an important role in actin filament arrangement [40].

B and T lymphocytes express the most DOCK8 [42]. Hence, DOCK8-mediated HIES is associated with immunological effects that occur at multiple stages in T- and B-cell development. Findings include reduced CD8^+ ^T-cell stimulation and clonal proliferation [43]. Furthermore, there is a paucity of naive CD8^+^CD45RA^+^CCR7^+ ^circulating T cells; to a lesser degree, there is a decline in CD8^+^CD45RA^-^CCR7^-/+ ^memory T cells and an increase in a senescent population of effector memory CD45RA^+ ^(T_EMRA_) cells with the phenotype CD8^+^CD57^+^CX3CR1^+^CD27^-^CD28^-^CD127^- ^[42]. Some patients with this condition may have diminished T_H_17 cell functional responses; however, these findings do not appear as prominent as in AD-HIES [44]. A mouse model with a DHR2-deficient, homozygous, null DOCK8 allele strain suggests that the most profound T-cell effects are on CD4^+ ^and CD8^+ ^long-lived memory T-cell survival [45]. In a mouse model, B-cell effects include an attenuated ability to form marginal zone B cells and to maintain germinal center B cells with resultant defects in high-affinity antibody generation [45,46]. Engelhardt et al[40] postulate that derangement in cell cytoskeletal organization has the potential to affect immunologic synapse formation.

## Tyrosine Kinase 2 Signaling

**Figure 3:**
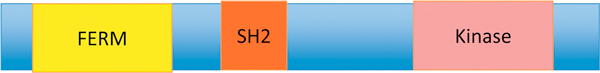
**Organization of the TYK2 protein**.

Tyrosine kinase 2 (TYK2) is in the family of Janus kinase molecules, of which there are 4 known in humans. *TYK2 *is located on chromosome 19p13.2 [47-49]. TYK2 transduces signals transmitted from type I interferon receptors (interferon α and β), cytokine receptors sharing IL-12Rβ1 subunit (IL-12, IL-23), cytokine receptors sharing a gp 130 subunit (IL-6, IL-10, CNTF, LIF, IL-11), and IL-13 [50-58]. The TYK2 protein includes a FERM domain (important in localizing the protein to the plasma membrane),[59,60] an SH2 domain (important in modulating regulation of intracellular signaling cascades),[61-64] and a kinase domain (important in phosphorylation of target proteins, such as STAT proteins, which can cause the affected protein to change cellular location or can affect association with other proteins)[65] (Figure 3).

Homozygous deletion mutations have been identified originating in the FERM domain and before the kinase domain in 2 different individuals. In vitro human TYK2-deficient cells, from the FERM origination mutant cells, stimulated with interferon α, did not lead to tyrosine phosphorylation of JAK1, STAT1, STAT2, STAT3, and STAT4 [66]. Furthermore, stimulation by IL-6 and IL-10 did not increase SOCS3 levels, a repressor of STAT3 [66]. TYK2 deficiency is associated with decreased T_H_1 differentiation and increased T_H_2 differentiation postulated to be TYK2-regulated IL-12 effects [66]. T-cell replenishment with TYK2 restored IL-12 and interferon α signaling [66]. In a mouse model, impaired TYK2 signaling supported the human impaired T_H_1 response findings and demonstrated diminished interferon γ secretion by IL-12-stimulated splenocytes [67]. Impaired T_H_1 findings might give further clues to susceptibility to mycobacterial infections in this condition. The mouse model also introduced the notion of impaired IL-17 responses in TYK2-deficient mice by IL-23-stimulated splenocytes, which might explain impaired fungal responses identified in this condition [67].

## Clinical Presentation of AD-HIES

AD-HIES typically presents in the neonate stage marked by papular or pustular rashes that mimic acne, eosinophilic dermatitis, or eczema. Lesions typically begin on the head and scalp before progressing over the rest of the body and frequently become superinfected with *Staphylococcus aureus*, resulting in recurrent abscess formation. These recurrent abscesses are pus filled but lack typical inflammatory responses like warmth, erythema, and pain [68,69]. Viral skin infections are rare in AD-HIES.

Within the first years of life, these patients also get frequent pneumonias with *S. aureus. Streptococcus pneumoniae *and *Haemophilus influenzae *are the next most common pathogens. Symptoms include purulent sputum, but fever is typically absent. Infection may lead to lung parenchyma destruction, bronchiectasis, and pneumatocele formation in the majority of affected subjects [68,69].

Once structural changes occur in the lung, susceptibility to invasive mycotic infections can ensue as reported by Vinh et al.[70] Forty-four percent of patients with structural damage in this cohort contracted at least one such infection [70]. In patients with pneumatoceles and bronchiectasis, *Pseudomonas aeruginosa *and *Aspergillus fumigatus *are frequently implicated pathogens. A study by Melia et al[71] concluded that subjects with lung parenchyma damage have increased risk of infection by nontuberculous mycobacteria. Rarely, infection progresses to massive hemoptysis [72].

**Figure 4:**
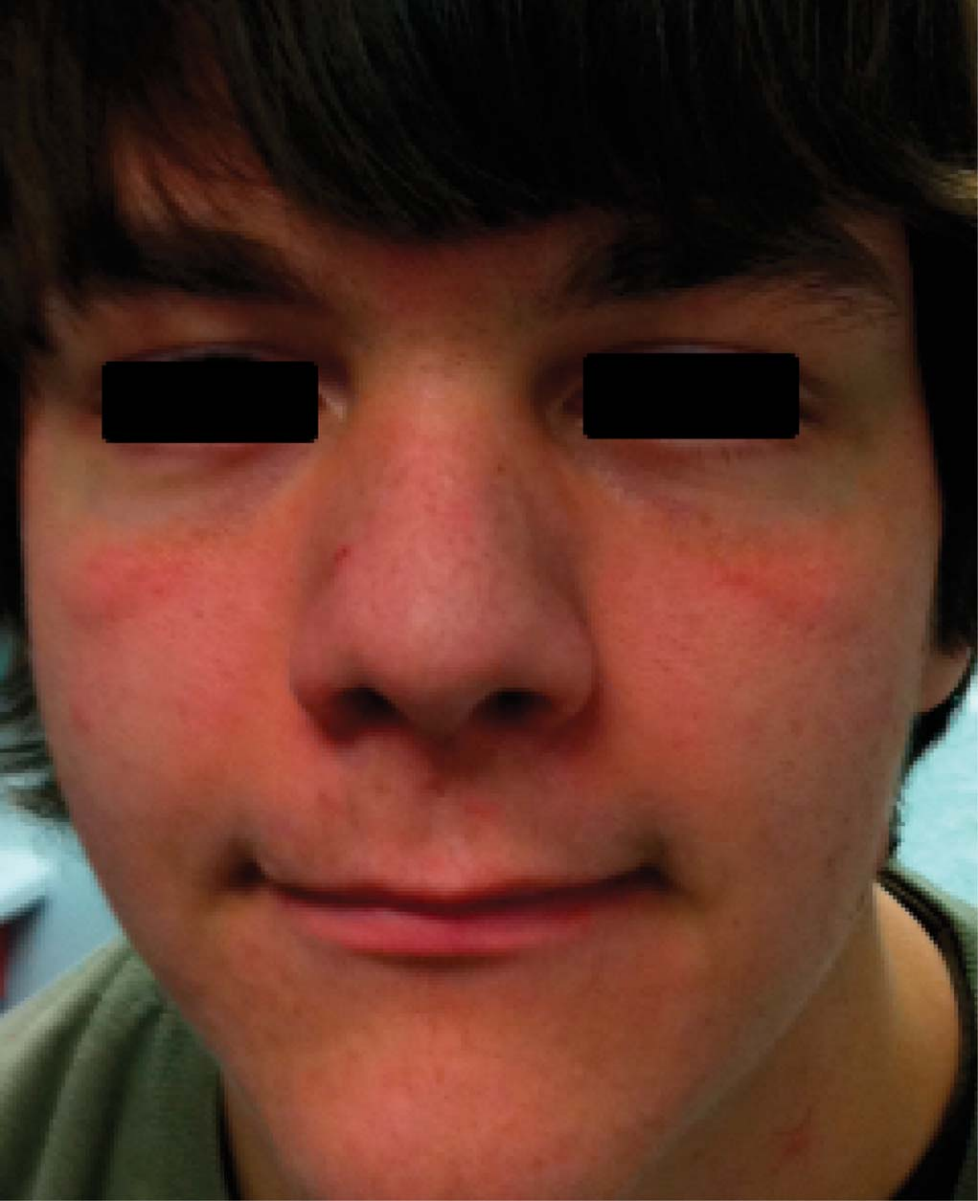
**Facial features in AD-HIES**.

**Figure 5:**
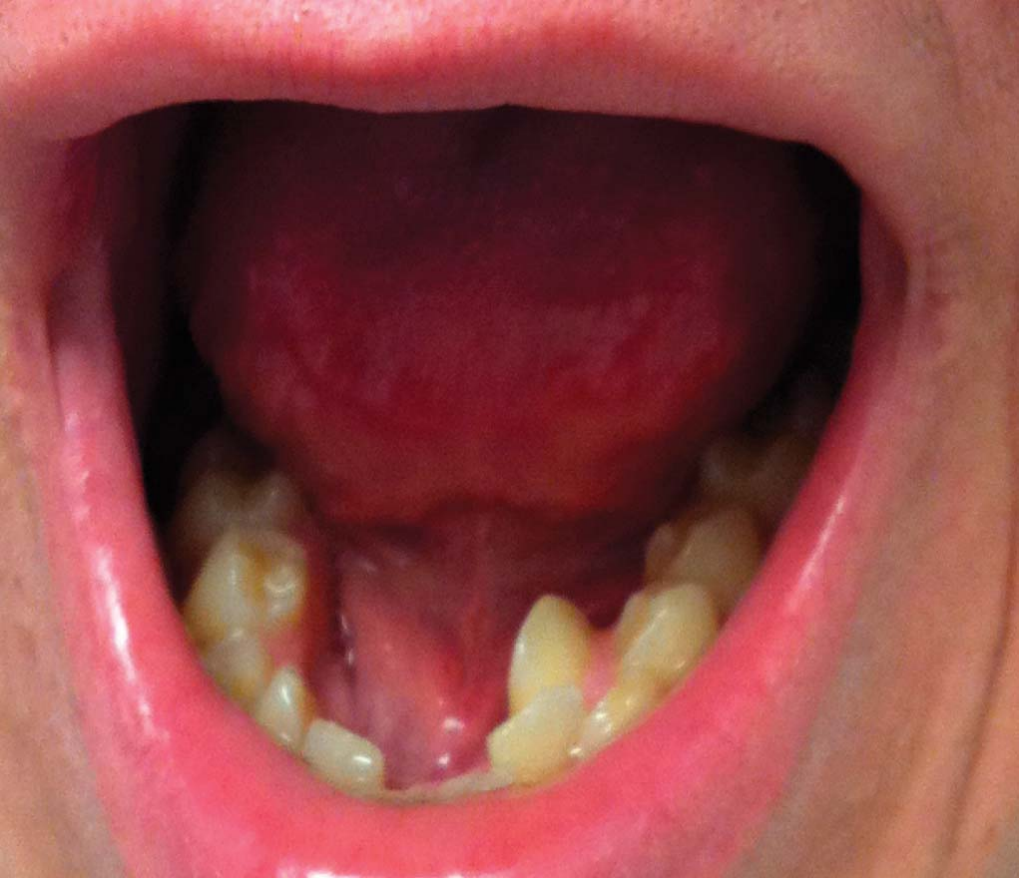
**Retention of primary teeth in a patient with AD-HIES**.

AD-HIES patients have prominent oral and facial findings that develop from childhood through the teenage years. Findings include facial asymmetry, prominent forehead, broad nose, deep eyes, rough facial skin, and retention of primary teeth, which may cause impaction or a double row of teeth (Figures 4, 5).

On the hard palate, subjects may have fibrotic bridges that typically run anterior to posterior, which may or may not have associated grooves or clefts. The tongue surface has prominent fissures and a deep midline cleft anterior to the circumvallate papillae is common. This pattern of fissures can also be seen on the lips, cheeks, and mucosa, with prominent keratotic striations [68]. Mucocutaneous candidiasis is also common in affected patients [69]. Salivary gland defects in antimicrobial proteins, β-defensin-2 and histatins, have been attributed to reduced candidacidal responses [24].

**Figure 6:**
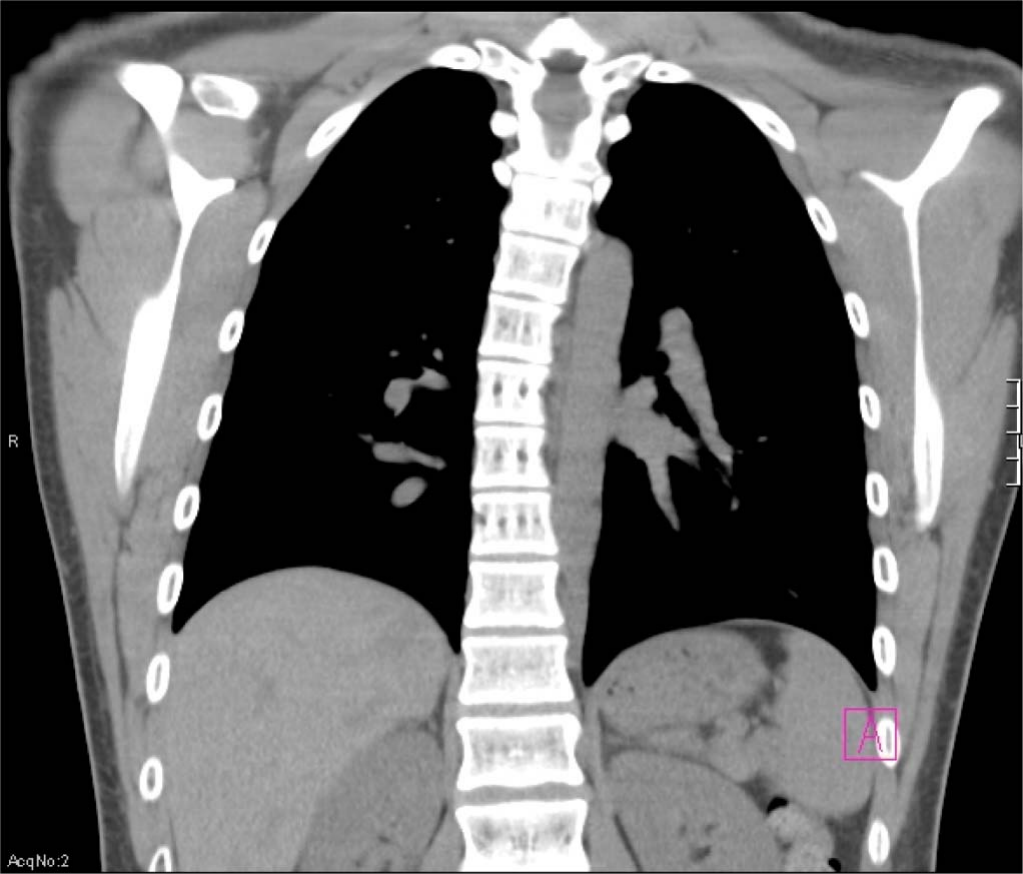
**Scoliosis in a patient with AD-HIES**.

From adolescence onward, the skeletal manifestations of this disorder become prominent. About 75% of HIES teenagers develop scoliosis, which can be severe enough to necessitate rod implantation (Figure 6).

Severe osteopenia and osteoporosis is common in affected subjects. Frequent fractures of long bones and ribs are reported in approximately 50% of patients. These fractures are not necessarily associated with osteopenia. In the third and fourth decade, degenerative joint disease, often of the spine, can become debilitating and requires surgical correction [68].

Vascular manifestations of AD-HIES vary in location and severity. Aortic aneurysm rupture has been reported [73,74]. Coronary artery dilation and tortuosity appears common [75]. Specifically, dilation of the left anterior descending coronary artery followed by right coronary artery involvement with prevalences of 51% and 25% was reported by Freeman et al.[75] In contrast, tortuosity appeared more common in the right coronary artery versus the left anterior descending [75]. In this cohort, the findings suggested vessel dilation and tortuosity prevalence increased with age and occurred more often in men [75]. Coronary arteries typically lack evidence of atherosclerotic damage [76]. Other reported vascular abnormalities include patent ductus venosus, pseudoaneurysms, and superior vena cava syndrome [74]. Central nervous system (CNS)-reported abnormalities include vasculitis, leading to a right parietal infarction and thrombosis of the left posterior inferior cerebellar artery [74].

Gastrointestinal (GI) manifestations of AD-HIES are rare. One case report described a 26-year-old man with sigmoid diverticulitis that progressed to a pelvic abscess. Resolution required drainage, intravenous antibacterial and antifungal agents, and sigmoid colectomy for successful treatment [77]. There have been 2 published reports of spontaneous colonic perforations: one occurred in an 18-year-old man who developed cecal perforation and the other involved an 8-year-old girl who developed transverse colon perforation [78,79]. Three cases of histoplasmosis of the GI tract have been reported. Two occurred in teenage patients thought to have Crohn disease before pathology revealed histoplasmosis and the other occurred in an adult with recurrent colonic histoplasmosis [80,81].

AD-HIES patients also have an increased risk of developing lymphomas. This risk may be as high as 259-fold over the general population [82]. A review of 23 subjects with lymphoma identified 6 with T-cell lymphomas, 13 with B-cell lymphomas, and 4 with classic Hodgkin lymphomas. Of the B-cell lymphomas, pathologic analysis revealed 6 as diffuse large B-cell lymphomas and 3 as Burkitt lymphomas [83,84]. Other reported lymphomas include peripheral T-cell lymphomas and mantle cell lymphomas [84].

## Clinical Presentation of DOCK8 AR-HIES

Patients with DOCK8 immunodeficiency syndrome (DIDS), the most common form of AR-HIES, have a slightly different presentation from that described above (Table 1). The severe eczema and recurrent skin infections or abscesses caused by *S. aureus *are still prominent, but affected subjects have a higher rate of viral infections, which include molluscum contagiosum, herpes simplex virus, and varicella (Zhang, Englehardt). Food and environmental allergies are common [40,43]. Asthma, eosinophilic esophagitis, and anaphylaxis have also been variably reported [40,43]. Recurrent upper respiratory infections, such as otitis media, otitis externa, and sinusitis, are common, whereas mastoiditis and recurrent croup are rare [43]. Although frequent pneumonias are still common in this group, they typically do not result in pneumatoceles. Implicated lung pathogens include *S. pneumoniae, Haemophilus influenza, Pneumocystis jiroveci, Proteus mirabilis, P. aeruginosa, Cryptococcus*, respiratory adenovirus, and respiratory syncytial virus [43,85]. Mortality from sepsis is much higher in the DIDS patients.

**Table 1:** Distinguishing Features Comparing AD-HIES With DIDS and TYK2 HIES

Clinical Finding	AD-HIES	DIDS AR-HIES	TYK2 AR-HIES
Viral skin infections	-	+++	++
Pneumatoceles	++	-	-
Recurrent pneumonia	++	++	++
Sepsis mortality	+	+++	+++
Facial dysmorphic features	+++	-	-
Primary tooth retention	+++	-	-
Scoliosis	++	-	-
Neurologic manifestations	+	+++	++
Cerebral vascular abnormalities	+	++	++
Food allergies	-	+++	-
Environmental allergies	-	++	
Decreased IgM	-	++	-
Atypical mycobacterial infections	-	-	++
Malignancy	+	+++	?
Lymphoma	+	+	?

The typical facial presentation of a broad nose, prominent forehead, and facial asymmetry, as well as retention of primary teeth, is absent. Scoliosis is rare in this subset. Mucocutaneous candidiasis is common in DIDS.

There is an increase in neurologic manifestations in DIDS, including facial paralysis, hemiplegia, and CNS vasculitis [85]. Reported CNS infections include JC virus-associated progressive multifocal leukoencephalopathy, and meningitis associated with Cryptococcus and *Haemophilus influenza*[40,43]. Cerebral vascular abnormalities are more common in this group than in AD-HIES [74]. In the CNS, ruptured cerebral aneurysm and subarachnoid hemorrhage have resulted in death before clinical diagnosis, but to our knowledge, this has only been reported twice in the literature in AR-HIES [85]. The discovery of CNS vascular abnormalities may occur incidentally after a stroke or hemiplegia and precipitating features include under-perfusion of large arteries and diminished basal cerebral artery caliber. Aneurysms of the ascending aorta and pericarditis are described affecting the cardiovascular system [74].

In the GI tract, salmonella enteritis and giardiasis have been reported. Mortality from malignancy is far higher in DIDS than in AD-HIES. Skin-originating cancers are more common and include fatal metastatic squamous cell carcinoma and fatal cutaneous T-cell lymphoma-leukemia [43]. Burkitt lymphoma has been rarely reported [40]. Autoimmune hemolytic anemia has also been reported [40].

DIDS laboratory findings can reveal, in addition to elevated IgE and eosinophilia seen in AD-HIES, low absolute lymphocyte counts, low total T-cell counts, with low CD4^+ ^and low CD8^+ ^counts but normal CD4^+ ^to CD8^+ ^ratio [40,43]. Neutrophil and monocyte numbers are usually normal, whereas natural killer cell and B cell levels are variable [40,43]. IgG levels can be normal or elevated, IgA levels are variable, IgE levels are typically elevated, and IgM levels are usually low [40,43].

## Clinical Presentation of TYK2 AR-HIES

Two patients with TYK2 deficiency have been reported [66,86]. The 2 patients had homozygous mutations in *TYK2*, but in different locations of the gene. Furthermore, the patients were of different ethnic backgrounds and had varied clinical symptoms. Both patients lacked features typical to AD-HIES, such as skeletal or dental alterations and both patients had susceptibility to intracellular *Mycobacterium bovis *after Bacille Calmette-Geurin (BCG) vaccination, but with variable severity. Both had episodes of cutaneous herpes infection but with variable severity.

The patient with more severe infections was of Turkish descent, had an early termination codon in TYK2 at amino acid position 767 caused by a 9-base pair DNA deletion in exon 16, had mildly elevated IgE (to a maximum of 218 IU/mL), no atopy, asthma, skin candidiasis, boils, folliculitis, or cold abscesses. This patient developed 2 disseminated infections with intracellular organisms. Specifically, 6 months after vaccination with BCG, he was noted to have axillary lymphadenopathy at age 8 months. This infection relapsed after treatment with isoniazid and became generalized at age 21 months, affecting the cervical, axillary, and inguinal lymph nodes bilaterally. Laboratory assessment during chronic infection demonstrated normal nitroblue tetrazolium test, normal serum IgG, IgM, IgA, and mildly elevated IgE. He had no relapse after 18 months of treatment. The patient was subsequently infected at age 8, with the intracellular organism *Brucella *spp after ingestion of unpasteurized cheese. The patient was treated with a 6-week antibiotic course and was found to have neurobrucellosis and pneumonia 1 month later. The patient was severely debilitated after this episode with left temporal, occipital, and bilateral parietal brain infarcts accompanied by sensorineural hearing loss and residual cognitive impairment. At age 11, he had an episode of herpes zoster involving the right maxillary branch of the trigeminal nerve [86].

The patient with more mild infections was of Japanese descent, had an early termination codon at amino acid position 90 caused by a frame shift mutation at DNA coding regions 70 to 89, had elevated IgE (2100 IU/mL), and susceptibility to viruses, fungi, mycobacteria, and intracellular and extracellular bacteria. He was noted to have atopic dermatitis-like rash in the first month of life, skin abscesses, oral candidiasis, recurrent otitis media, sinusitis, pneumonias, molluscum contagiosum, and herpes simplex infection of the skin and mucosa. He was also noted to develop 2 infections with intracellular infections. The first infection was to BCG at age 22 months. The second infection was to non-typhi salmonella gastroenteritis at age 15. Peripheral blood laboratory assessment revealed normal quantitative T, B, and natural killer cells and normal neutrophil function. In vitro peripheral blood mononuclear cell studies from the patient demonstrated higher basal class I HLA expression, complete defects in type I interferon responses, and failure to make interferon γ after IL-12 stimulation. In vitro studies also revealed blunted IL-6 and IL-10 responses with either absent or attenuated feedback inhibition through suppressor of cytokine signaling (SOCS)3 expression after cytokine stimulation. In vitro studies also revealed increased IL-5 and IL-13 when naive CD4^+ ^T cells were stimulated with IL-2 and anti-CD3 [66].

## Diagnosis

Diagnosis of HIES is based on clinical and laboratory findings originally described in a NIH HIES scoring criteria consisting of 21 features [87]. Although the NIH scale is sensitive for the presence of HIES, it is not specific for the underlying mutation [88]. However, analysis of mutations focused on groups of mutations specifically localized to the STAT3 SH2- and DNA-binding domains, the most common mutation regions, identified that subjects with SH2 domain mutations had a statistically significant increase in scoliosis at a younger age, high palate, increased intertalar distance, and increased otitis and sinusitis [89]. The same cohort had more deaths from infection in subjects with DNA-binding domain mutations versus subjects with SH2-binding domain mutation [89].

The features identified in the NIH scale that are most suggestive of a *STAT3 *mutation include: abscesses of internal organs, other severe infections, pneumatoceles, nail/mucocutaneous candidiasis, bone fractures with minimal trauma, scoliosis, and a family history of HIES [88]. A subject with a family history of HIES and a score > 40 points is highly likely to have a HIES genotype, but unlikely if they score less than 20 points [90]. Attempts to refine more sensitive and specific criteria for the diagnosis of specific forms of HIES are ongoing [88].

Revised clinical guidelines for the diagnosis of STAT3 mutant HIES have been proposed by Woellner et al.[7] A possible diagnosis can be made with IgE levels ≥1000 IU/mL plus a weighted score more than 30 based on 5 clinical findings: recurrent pneumonias, newborn rash, pathologic bone fractures, facial appearance, and high palate. Probable diagnosis would include the above plus a family history of HIES or lack of T_H_17 cells, and a definitive diagnosis would require a known *STAT3 *mutation [7]. While these clinical predictors have been found useful in better classifying patients with *STAT3 *mutations, the features unique to the AR-HIES secondary to *DOCK8 *were not addressed.

*STAT3 *mutation analysis is currently available commercially and used in the research setting, given the large size of the gene and expense of this endeavor. The use of high resolution polymerase chain reaction-based DNA-melting assays to identify and screen patients for *STAT3 *mutations are under investigation [91]. This assay is quicker and cheaper than genomic sequencing and screens for sequence variants by DNA melting point changes. The variants can be subsequently confirmed through targeted sequence analysis, thereby lowering the cost in relation to complete genome sequencing [91]. This technique is reported to have 100% sensitivity in a cohort of 16 patients for the identification of *STAT3 *mutations in AD-HIES patients and is expected to identify such mutations in 90% of AD-HIES patients [91]. To date, there is no phenotype/genotype relationship among the numerous *STAT3 *mutations identified [7].

T-cell receptor excision circle (TREC) analysis in newborn screening to detect severe combined immune deficiency identified 3 consanguineous siblings with DIDS to have low TRECs in one sibling and undetectable TRECs in the older siblings aged 4 and 6 [92]. These findings raise the possibility for early detection of DIDS and add this condition to the differential diagnosis for absent or low TRECs on newborn screening.

Commercially available tests for diagnosis of DOCK8 and TYK2 deficiency are limited. DIDS diagnostic considerations include assessment for low serum IgM, low T-cell numbers, and low T-cell proliferative responses. DOCK8 and *TYK2 *sequencing is available at specialized research laboratories.

## Treatment

The importance of early recognition and treatment of *S. aureus *and fungal infections is essential as these patients often display fewer of the traditional findings and symptoms associated with these infections [93]. Although skin infections are often treated with anti-staphylococcal antibiotic therapy, antibacterial ointments, dilute bleach, or chlorinated water, recrudescence is one of the classic signs of AD-HIES [93,94]. Viral skin infections in DIDS are challenging and variably responsive to anti-viral therapy. Respiratory infections can lead to lung parenchymal damage; hence, eradication of pulmonary infections is important. Targeted therapy based on culture results could be considered given the risk for antimicrobial resistance with repeated antibiotic exposure. A high index of suspicion for intracellular organism infections could be considered in infections associated with TYK2 deficiency.

Hematopoietic cell transplantation (HCT) has been used in treating both the autosomal recessive and autosomal dominant forms of the disease. Limited information exists regarding long-term outcome; however, many experts recommend transplantation for DIDS, as long-term outcome without transplantation is poor owing to the risk for fatal infections, malignancy risk and CNS infarction, and bleeding [95]. Successful long-term improvement, despite mixed donor chimerism, has been reported in DIDS [95]. Other reports have been mixed. One case study reported successful engraftment with the CD3^+ ^and CD15^+ ^cells being identified as donor 21 days after HCT. After discharge, the subject did not show evidence of any skin infections. Unfortunately, long-term follow-up was unable to be performed as the patient was hospitalized and died 58 days after transplantation due to *Klebsiella pneumoniae *bacteremia. The authors speculate that the transplant recipient's congenital asplenia contributed to morbidity and mortality [96]. Another case series reported the results of HCT on 2 patients with AR-HIES. Although one patient had a complicated transplant course with multiple ulcerative lesions, brain abscesses, and an acute EBV infection after recovery and successful donor stem cell engraftment, both patients demonstrated significant clinical improvement with resolution of their molluscum contagiosum lesions and continued absence of cutaneous viral infections 2 and 4 years after transplantation. Laboratory assessment of the patients' immune function was normal with the exception of low IgA levels in both patients [97].

Some experts recommend using HCT in AD-HIES patients with malignancy because other disease-related effects can be managed without HCT. The role of HCT in the treatment of AD-HIES is less consistent than reports on patients with DIDS. One case series reported 2 subjects with AD-HIES and non-Hodgkin lymphoma who underwent allogeneic HCT with successful engraftment, and complete donor chimerism was confirmed by CD3^+ ^cell analysis. After transplantation, both subjects demonstrated normalized IgE levels, which remained normal for 14 and 10 years after transplantation, respectively. In addition, neither subject has had recurrent infections requiring hospitalization, and both have been free of skin infections [97]. Osteoporosis, which was present in one subject, resolved after transplantation. Furthermore, the T_H_17 counts were normal [4]. This is in contrast with an earlier report noting the failure of HCT to correct HIES in a patient who relapsed 4 years after transplantation; however, the mutation status of the patient was not addressed in the original article [98].

## Competing interests

The authors declare that they have no competing interests.

## References

[B1] Choi JY Li WL Kouri RE Yu J Kao FT Ruano G Assignment of the acute phase response factor (APRF) gene to 17q21 by microdissection clone sequencing and fluorescence in situ hybridization of a P1 clone Genomics 1996 5 264 265 10.1006/geno.1996.0556 8921406

[B2] Kisseleva T Bhattacharya S Braunstein J Schindler CW Signaling through the JAK/STAT pathway, recent advances and future challenges Gene 2002 5 1 24 10.1016/S0378-1119(02)00398-0 12039028

[B3] Levy DE Darnell JE Jr Stats: transcriptional control and biological impact Nat Rev Mol Cell Biol 2002 5 651 662 12209125 10.1038/nrm909

[B4] Wegrzyn J Potla R Chwae YJ Sepuri NBV Zhang Q Function of mitochondrial Stat3 in cellular respiration Science 2009 5 793 797 10.1126/science.1164551 19131594 PMC2758306

[B5] Pelletier S Duhamel F Coulombe P Popoff MR Meloche S Rho family GTPases are required for activation of Jak/STAT signaling by G protein coupled receptors Mol Cell Biol 2003 5 1316 1333 10.1128/MCB.23.4.1316-1333.2003 12556491 PMC141129

[B6] Heim MH Sehgal PB, Levy DE, Hirano T The STAT protein family Signal Transducers and Activators of Transcription (STATs). Activation and Biology 2003 Dordrecht, The Netherlands: Kluwer Academic Publishers 11 26

[B7] Woellner C Gertz EM Schaffer AA Lagos M Perro M Mutations in STAT3 and diagnostic guidelines for hyper-IgE syndrome J Allergy Clin Immunol 2010 5 424 432 e428 10.1016/j.jaci.2009.10.059 20159255 PMC2878129

[B8] Mintz R Garty BZ Meshel T Marcus N Katanov C Cohen-Hillel E Ben-Baruch A Reduced expression of chemoattractant receptors by polymorphonuclear leukocytes in Hyper IgE Syndrome patients Immunol Lett 2010 5 97 106 10.1016/j.imlet.2009.12.006 20005258

[B9] Rossi D Zlotnik A The biology of chemokines and their receptors Annu Rev Immunol 2000 5 217 242 10.1146/annurev.immunol.18.1.217 10837058

[B10] Zeilhofer HU Schorr W Role of interleukin-8 in neutrophil signaling Curr Opin Hematol 2000 5 178 182 10.1097/00062752-200005000-00009 10786656

[B11] Mukaida N Interleukin-8: an expanding universe beyond neutrophil chemotaxis and activation Int J Hematol 2000 5 391 398 11197203

[B12] Baggiolini M Loetscher P Moser B Interleukin-8 and the chemokine family Int J Immunopharmacol 1995 5 103 108 10.1016/0192-0561(94)00088-6 7657403

[B13] Murphy PM Neutrophil receptors for interleukin-8 and related CXC chemokines Semin Hematol 1997 5 311 318 9347581

[B14] Chuntharapai A Kim KJ Regulation of the expression of IL-8 receptor A/B by IL-8: possible functions of each receptor J Immunol 1995 5 2587 2594 7650389

[B15] Richardson RM Pridgen BC Haribabu B Ali H Snyderman R Differential cross-regulation of the human chemokine receptors CXCR1 and CXCR2. Evidence for time-dependent signal generation J Biol Chem 1998 5 23830 23836 10.1074/jbc.273.37.23830 9726994

[B16] Green SP Chuntharapai A Curnutte JT Interleukin-8 (IL-8), melanoma growth-stimulatory activity, and neutrophil-activating peptide selectively mediate priming of the neutrophil NADPH oxidase through the type A or type B IL-8 receptor J Biol Chem 1996 5 25400 25405 10.1074/jbc.271.41.25400 8810307

[B17] Feniger-Barish R Yron I Meshel T Matityahu E Ben-Baruch A IL-8-induced migratory responses through CXCR1 and CXCR2: association with phosphorylation and cellular redistribution of focal adhesion kinase Biochemistry 2003 5 2874 2886 10.1021/bi026783d 12627953

[B18] Feniger-Barish R Ran M Zaslaver A Ben-Baruch A Differential modes of regulation of cxc chemokine-induced internalization and recycling of human CXCR1 and CXCR2 Cytokine 1999 5 996 1009 10.1006/cyto.1999.0510 10623425

[B19] Richardson RM Ali H Pridgen BC Haribabu B Snyderman R Multiple signaling pathways of human interleukin-8 receptor A. Independent regulation by phosphorylation J Biol Chem 1998 5 10690 10695 10.1074/jbc.273.17.10690 9553132

[B20] Hammond ME Lapointe GR Feucht PH Hilt S Gallegos CA IL-8 induces neutrophil chemotaxis predominantly via type I IL-8 receptors J Immunol 1995 5 1428 1433 7636208

[B21] Van Damme J Wuyts A Froyen G Van Coillie E Struyf S Granulocyte chemotactic protein-2 and related CXC chemokines: from gene regulation to receptor usage J Leukoc Biol 1997 5 563 569 9365109 10.1002/jlb.62.5.563

[B22] Zlotnik A Yoshie O Nomiyama H The chemokine and chemokine receptor superfamilies and their molecular evolution Genome Biol 2006 5 243 10.1186/gb-2006-7-12-243 17201934 PMC1794421

[B23] Minegishi Y Saito M Nagasawa M Takada H Hara T Molecular explanation for the contradiction between systemic Th17 defect and localized bacterial infection in hyper-IgE syndrome J Exp Med 2009 5 1291 1301 10.1084/jem.20082767 19487419 PMC2715068

[B24] Conti HR Baker O Freeman AF Jang WS Holland SM New mechanism of oral immunity to mucosal candidiasis in hyper-IgE syndrome Mucosal Immunol 2011 5 448 455 10.1038/mi.2011.5 21346738 PMC3119375

[B25] Siegel AM Heimall J Freeman AF Hsu AP Brittain E A critical role for STAT3 transcription factor signaling in the development and maintenance of human T cell memory Immunity 2011 5 806 818 10.1016/j.immuni.2011.09.016 22118528 PMC3228524

[B26] Ozaki K Spolski R Feng GC Qi CF Cheng J A critical role for IL-21 in regulating immunoglobulin production Science 2002 5 1630 1634 10.1126/science.1077002 12446913

[B27] Wood N Bourque K Donaldson DD Collins M Vercelli D Goldman SJ Kasaian MT IL-21 effects on human IgE production in response to IL-4 or IL-13 Cell Immunol 2004 5 133 145 10.1016/j.cellimm.2005.01.001 15919378

[B28] Avery DT Deenick EK Ma CS Suryani S Simpson N B cellintrinsic signaling through IL-21 receptor and STAT3 is required for establishing long-lived antibody responses in humans J Exp Med 2010 5 155 171 10.1084/jem.20091706 20048285 PMC2812540

[B29] Speckmann C Enders A Woellner C Thiel D Rensing-Ehl A Reduced memory B cells in patients with hyper IgE syndrome Clin Immunol 2008 5 448 454 10.1016/j.clim.2008.08.002 18835223

[B30] Meyer-Bahlburg A Renner ED Rylaarsdam S Reichenbach J Schimke LF Heterozygous signal transducer and activator of transcription 3 mutations in hyper-IgE syndrome result in altered B-cell maturation J Allergy Clin Immunol 2012 5 559 562 562 e551-552 10.1016/j.jaci.2011.09.017 22030463

[B31] Sekhsaria V Dodd LE Hsu AP Heimall JR Freeman AF Plasma metalloproteinase levels are dysregulated in signal transducer and activator of transcription 3 mutated hyper-IgE syndrome J Allergy Clin Immunol 2011 5 1124 1127 10.1016/j.jaci.2011.07.046 21872914 PMC4074374

[B32] Zhou H Newnum AB Martin JR Li P Nelson MT Osteoblast/osteocyte-specific inactivation of Stat3 decreases load-driven bone formation and accumulates reactive oxygen species Bone 2011 5 404 411 10.1016/j.bone.2011.04.020 21555004

[B33] Lufei C Ma J Huang G Zhang T Novotny-Diermayr V Ong CT Cao X GRIM-19, a death-regulatory gene product, suppresses Stat3 activity via functional interaction EMBO J 2003 5 1325 1335 10.1093/emboj/cdg135 12628925 PMC151078

[B34] Griggs BL Ladd S Saul RA DuPont BR Srivastava AK Dedicator of cytokinesis 8 is disrupted in two patients with mental retardation and developmental disabilities Genomics 2008 5 195 202 10.1016/j.ygeno.2007.10.011 18060736 PMC2245991

[B35] Zhang Q Davis JC Dove CG Su HC Genetic, clinical, and laboratory markers for DOCK8 immunodeficiency syndrome Dis Markers 2010 5 131 139 10.1155/2010/972591 21178272 PMC3835385

[B36] Raptis L Arulanandam R Geletu M Turkson J The R(h)oads to Stat3: Stat3 activation by the Rho GTPases Exp Cell Res 2011 5 1787 1795 10.1016/j.yexcr.2011.05.008 21619876 PMC3129747

[B37] Debidda M Wang L Zang H Poli V Zheng Y A role of STAT3 in Rho GTPase-regulated cell migration and proliferation J Biol Chem 2005 5 17275 17285 10.1074/jbc.M413187200 15705584

[B38] Faruqi TR Gomez D Bustelo XR Bar-Sagi D Reich NC Rac1 mediates STAT3 activation by autocrine IL-6 Proc Natl Acad Sci USA 2001 5 9014 9019 10.1073/pnas.161281298 11470914 PMC55365

[B39] Simon AR Vikis HG Stewart S Fanburg BL Cochran BH Guan KL Regulation of STAT3 by direct binding to the Rac1 GTPase Science 2000 5 144 147 10.1126/science.290.5489.144 11021801

[B40] Engelhardt KR McGhee S Winkler S Sassi A Woellner C Large deletions and point mutations involving the dedicator of cytokinesis 8 (DOCK8) in the autosomal-recessive form of hyper-IgE syndrome J Allergy Clin Immunol 2009 5 1289 1302 e1284 10.1016/j.jaci.2009.10.038 20004785 PMC2818862

[B41] Cote JF Vuori K GEF what? Dock180 and related proteins help Rac to polarize cells in new ways Trends Cell Biol 2007 5 383 393 10.1016/j.tcb.2007.05.001 17765544 PMC2887429

[B42] Randall KL Chan SS Ma CS Fung I Mei Y DOCK8 deficiency impairs CD8 T cell survival and function in humans and mice J Exp Med 2011 5 2305 2320 10.1084/jem.20110345 22006977 PMC3201196

[B43] Zhang Q Davis JC Lamborn IT Freeman AF Jing H Combined immunodeficiency associated with DOCK8 mutations N Engl J Med 2009 5 2046 2055 10.1056/NEJMoa0905506 19776401 PMC2965730

[B44] Al Khatib S Keles S Garcia-Lloret M Karakoc-Aydiner E Reisli I Defects along the T(H)17 differentiation pathway underlie genetically distinct forms of the hyper IgE syndrome J Allergy Clin Immunol 2009 5 342 348 348 e341-345 10.1016/j.jaci.2009.05.004 19577286 PMC2828264

[B45] Lambe T Crawford G Johnson AL Crockford TL Bouriez-Jones T DOCK8 is essential for T-cell survival and the maintenance of CD8(+) T-cell memory Eur J Immunol 2011 5 3423 3535 10.1002/eji.201141759 21969276 PMC3517112

[B46] Randall KL Lambe T Johnson AL Treanor B Kucharska E Dock8 mutations cripple B cell immunological synapses, germinal centers and long-lived antibody production Nat Immunol 2009 5 1283 1291 10.1038/ni.1820 19898472 PMC3437189

[B47] Firmbach-Kraft I Byers M Shows T Dalla-Favera R Krolewski JJ tyk2, prototype of a novel class of non-receptor tyrosine kinase genes Oncogene 1990 5 1329 1336 2216457

[B48] Trask B Fertitta A Christensen M Youngblom J Bergmann A Fluorescence in situ hybridization mapping of human chromosome 19: cytogenetic band location of 540 cosmids and 70 genes or DNA markers Genomics 1993 5 133 145 10.1006/geno.1993.1021 8432525

[B49] Krolewski JJ Lee R Eddy R Shows TB Dalla-Favera R Identification and chromosomal mapping of new human tyrosine kinase genes Oncogene 1990 5 277 282 2156206

[B50] Finbloom DS Winestock KD IL-10 induces the tyrosine phosphorylation of tyk2 and Jak1 and the differential assembly of STAT1 alpha and STAT3 complexes in human T cells and monocytes J Immunol 1995 5 1079 1090 7543512

[B51] Ho AS Wei SH Mui AL Miyajima A Moore KW Functional regions of the mouse interleukin-10 receptor cytoplasmic domain Mol Cell Biol 1995 5 5043 5053 7544437 10.1128/mcb.15.9.5043 PMC230751

[B52] Kopantzev Y Heller M Swaminathan N Rudikoff S IL-6 mediated activation of STAT3 bypasses Janus kinases in terminally differentiated B lineage cells Oncogene 2002 5 6791 6800 10.1038/sj.onc.1205815 12360405

[B53] Ihle JN Kerr IM Jaks and Stats in signaling by the cytokine receptor superfamily Trends Genet 1995 5 69 74 10.1016/S0168-9525(00)89000-9 7716810

[B54] Welham MJ Learmonth L Bone H Schrader JW Interleukin-13 signal transduction in lymphohemopoietic cells. Similarities and differences in signal transduction with interleukin-4 and insulin J Biol Chem 1995 5 12286 12296 10.1074/jbc.270.20.12286 7744881

[B55] Stahl N Boulton TG Farruggella T Ip NY Davis S Association and activation of Jak-Tyk kinases by CNTF-LIF-OSM-IL-6 beta receptor components Science 1994 5 92 95 10.1126/science.8272873 8272873

[B56] Narazaki M Witthuhn BA Yoshida K Silvennoinen O Yasukawa K Activation of JAK2 kinase mediated by the interleukin 6 signal transducer gp130 Proc Natl Acad Sci USA 1994 5 2285 2289 10.1073/pnas.91.6.2285 8134389 PMC43355

[B57] Du X Williams DA Interleukin-11: review of molecular, cell biology, and clinical use Blood 1997 5 3897 3908 9166826

[B58] Trinchieri G Pflanz S Kastelein RA The IL-12 family of heterodimeric cytokines: new players in the regulation of T cell responses Immunity 2003 5 641 644 10.1016/S1074-7613(03)00296-6 14614851

[B59] Chishti AH Kim AC Marfatia SM Lutchman M Hanspal M The FERM domain: a unique module involved in the linkage of cytoplasmic proteins to the membrane Trends Biochem Sci 1998 5 281 282 10.1016/S0968-0004(98)01237-7 9757824

[B60] Pearson MA Reczek D Bretscher A Karplus PA Structure of the ERM protein moesin reveals the FERM domain fold masked by an extended actin binding tail domain Cell 2000 5 259 270 10.1016/S0092-8674(00)80836-3 10847681

[B61] Marengere LE Pawson T Structure and function of SH2 domains J Cell Sci Suppl 1994 5 97 104 7883800 10.1242/jcs.1994.supplement_18.14

[B62] Pawson T Protein modules and signalling networks Nature 1995 5 573 580 10.1038/373573a0 7531822

[B63] Mayer BJ Baltimore D Signalling through SH2 and SH3 domains Trends Cell Biol 1993 5 8 13 10.1016/0962-8924(93)90194-6 14731533

[B64] Pawson T Schlessingert J SH2 and SH3 domains Curr Biol 1993 5 434 442 10.1016/0960-9822(93)90350-W 15335710

[B65] Manning G Plowman GD Hunter T Sudarsanam S Evolution of protein kinase signaling from yeast to man Trends Biochem Sci 2002 5 514 520 10.1016/S0968-0004(02)02179-5 12368087

[B66] Minegishi Y Saito M Morio T Watanabe K Agematsu K Human tyrosine kinase 2 deficiency reveals its requisite roles in multiple cytokine signals involved in innate and acquired immunity Immunity 2006 5 745 755 10.1016/j.immuni.2006.09.009 17088085

[B67] Ishizaki M Akimoto T Muromoto R Yokoyama M Ohshiro Y Involvement of tyrosine kinase-2 in both the IL-12/Th1 and IL-23/Th17 axes in vivo J Immunol 2011 5 181 189 10.4049/jimmunol.1003244 21606247

[B68] Freeman AF Domingo DL Holland SM Hyper IgE (Job's) syndrome: a primary immune deficiency with oral manifestations Oral Dis 2009 5 2 7 10.1111/j.1601-0825.2008.01463.x 19036057

[B69] Freeman AF Holland SM Clinical manifestations, etiology, and pathogenesis of the hyper-IgE syndromes Pediatr Res 2009 5 32R 37R 10.1203/PDR.0b013e31819dc8c5 19190525 PMC2919366

[B70] Vinh DC Sugui JA Hsu AP Freeman AF Holland SM Invasive fungal disease in autosomal-dominant hyper-IgE syndrome J Allergy Clin Immunol 2010 5 1389 1390 10.1016/j.jaci.2010.01.047 20392475 PMC2879472

[B71] Melia E Freeman AF Shea YR Hsu AP Holland SM Olivier KN Pulmonary nontuberculous mycobacterial infections in hyper-IgE syndrome J Allergy Clin Immunol 2009 5 617 618 10.1016/j.jaci.2009.07.007 19733303 PMC2740750

[B72] Ma BH Ng CS Lam RK Wan S Wan IY Lee TW Yim AP Recurrent hemoptysis with *Penicillium marneffei *and *Stenotrophomonas maltophilia *in Job's syndrome Can Respir J 2009 5 e50 e52 19707602 10.1155/2009/586919 PMC2734441

[B73] Netea MG Schneeberger PM de Vries E Kullberg BJ van der Meer JW Koolen MI Th1/Th2 cytokine imbalance in a family with hyper-IgE syndrome Neth J Med 2002 5 349 353 12572706

[B74] Yavuz H Chee R A review on the vascular features of the hyperimmunoglobulin E syndrome Clin Exp Immunol 2010 5 238 244 10.1111/j.1365-2249.2009.04044.x 19912258 PMC2819490

[B75] Freeman AF Avila EM Shaw PA Davis J Hsu AP Coronary artery abnormalities in hyper-IgE syndrome J Clin Immunol 2011 5 338 345 10.1007/s10875-011-9515-9 21494893 PMC4091041

[B76] Gharib AM Pettigrew RI Elagha A Hsu A Welch P Holland SM Freeman AF Coronary abnormalities in hyper-IgE recurrent infection syndrome: depiction at coronary MDCT angiography AJR Am J Roentgenol 2009 5 W478 W481 10.2214/AJR.09.2623 19933621 PMC4103904

[B77] Stover DG Freeman AF Wright PW Hummell DS Ness RM Diverticulitis in a young man with hyper-IgE syndrome South Med J 2010 5 1261 1263 10.1097/SMJ.0b013e3181fa5f0e 21037522 PMC3059239

[B78] Chen CM Lai HS Lin CL Hsieh KS Colon perforation in a patient with hyperimmunoglobulin E (Job's) syndrome J Pediatr Surg 1995 5 1479 1480 10.1016/0022-3468(95)90412-3 8786494

[B79] Hwang EH Oh JT Han SJ Kim H Colon perforation in hyperimmunoglobulin E syndrome J Pediatr Surg 1998 5 1420 1422 10.1016/S0022-3468(98)90025-2 9766371

[B80] Steiner SJ Kleiman MB Corkins MR Christenson JC Wheat LJ Ileocecal histoplasmosis simulating Crohn disease in a patient with hyperimmunoglobulin E syndrome Pediatr Infect Dis J 2009 5 744 746 10.1097/INF.0b013e31819b65e0 19633521

[B81] Cappell MS Manzione NC Recurrent colonic histoplasmosis after standard therapy with amphotericin B in a patient with Job's syndrome Am J Gastroenterol 1991 5 119 120 1986542

[B82] Leonard GD Posadas E Herrmann PC Anderson VL Jaffe ES Holland SM Wilson WH Non-Hodgkin's lymphoma in Job's syndrome: a case report and literature review Leuk Lymphoma 2004 5 2521 2525 10.1080/10428190400004463 15621772

[B83] Kumanovics A Perkins SL Gilbert H Cessna MH Augustine NH Hill HR Diffuse large B cell lymphoma in hyper-IgE syndrome due to STAT3 mutation J Clin Immunol 2010 5 886 893 10.1007/s10875-010-9452-z 20859667

[B84] Belada D Smolej L Stepankova P Kralickova P Freiberger T Diffuse large B-cell lymphoma in a patient with hyper-IgE syndrome: successful treatment with risk-adapted rituximab-based immunochemotherapy Leuk Res 2010 5 e232 e234 10.1016/j.leukres.2010.01.024 20226523

[B85] Renner ED Puck JM Holland SM Schmitt M Weiss M Autosomal recessive hyperimmunoglobulin E syndrome: a distinct disease entity J Pediatr 2004 5 93 99 10.1016/S0022-3476(03)00449-9 14722525

[B86] Kilic SS Hacimustafaoglu M Boisson-Dupuis S Kreins AY Grant AV Abel L Casanova JL A patient with tyrosine kinase 2 deficiency without hyper-IgE syndrome J Pediatr 2012 [Published ahead of print March 6, 2012] 10.1016/j.jpeds.2012.01.056 PMC3360808 22402565

[B87] Grimbacher B Schaffer AA Holland SM Davis J Gallin JI Genetic linkage of hyper-IgE syndrome to chromosome 4 Am J Hum Genet 1999 5 735 744 10.1086/302547 10441580 PMC1377980

[B88] Schimke LF Sawalle-Belohradsky J Roesler J Wollenberg A Rack A Diagnostic approach to the hyper-IgE syndromes: immunologic and clinical key findings to differentiate hyper-IgE syndromes from atopic dermatitis J Allergy Clin Immunol 2010 5 611 617 e611 10.1016/j.jaci.2010.06.029 20816194

[B89] Heimall J Davis J Shaw PA Hsu AP Gu W Welch P Holland SM Freeman AF Paucity of genotype-phenotype correlations in STAT3 mutation positive hyper IgE syndrome (HIES) Clin Immunol 2011 5 75 84 10.1016/j.clim.2011.01.001 21288777 PMC3065511

[B90] Ochs HD Smith CIE Puck J Primary Immunodeficiency Diseases: A Molecular and Genetic Approach 2007 New York, NY: Oxford University Press

[B91] Kumanovics A Wittwer CT Pryor RJ Augustine NH Leppert MF Rapid molecular analysis of the STAT3 gene in Job syndrome of hyper-IgE and recurrent infectious diseases J Mol Diagn 2010 5 213 219 10.2353/jmoldx.2010.090080 20093388 PMC2871728

[B92] Dasouki M Okonkwo KC Ray A Folmsbeel CK Gozales D Deficient T cell receptor excision circles (TRECs) in autosomal recessive hyper IgE syndrome caused by DOCK8 mutation: implications for pathogenesis and potential detection by newborn screening Clin Immunol 2011 5 128 132 10.1016/j.clim.2011.06.003 21763205 PMC4210456

[B93] Freeman AF Holland SM The hyper-IgE syndromes Immunol Allergy Clin North Am 2008 5 277 291 viii 10.1016/j.iac.2008.01.005 18424333 PMC2683262

[B94] Freeman AF Holland SM Clinical manifestations of hyper IgE syndromes Dis Markers 2010 5 123 130 10.1155/2010/580197 21178271 PMC3835387

[B95] Bittner TC Pannicke U Renner ED Notheis G Hoffmann F Successful long-term correction of autosomal recessive hyper-IgE syndrome due to DOCK8 deficiency by hematopoietic stem cell transplantation Klin Padiatr 2010 5 351 355 10.1055/s-0030-1265135 21058221

[B96] McDonald DR Massaad MJ Johnston A Keles S Chatila T Geha RS Pai SY Successful engraftment of donor marrow after allogeneic hematopoietic cell transplantation in autosomal-recessive hyper-IgE syndrome caused by dedicator of cytokinesis 8 deficiency J Allergy Clin Immunol 2010 5 1304 1305 e1303 10.1016/j.jaci.2010.07.034 20810158 PMC2998541

[B97] Gatz SA Benninghoff U Schutz C Schulz A Honig M Curative treatment of autosomal-recessive hyper-IgE syndrome by hematopoietic cell transplantation Bone Marrow Transplant 2011 5 552 556 10.1038/bmt.2010.169 20622910

[B98] Gennery AR Flood TJ Abinun M Cant AJ Bone marrow transplantation does not correct the hyper IgE syndrome Bone Marrow Transplant 2000 5 1303 1305 10.1038/sj.bmt.1702446 10871737

